# 

**Published:** 1850-04-01

**Authors:** 


### CONTENTS.

| | PAGE |
|---|---|
| The Passions | 142 |
| The Treatment of Insanity, Ancient and Modern | 164 |
| Moral State of Society | 183 |
| The Anatomy of the Human Brain | 197 |
| Insanity of Advanced Life | 212 |
| Annals of Medico-Psychology | 218 |
| Lectures on the Pathological Appearances in the Bodies of the Insane. No. I. By Dr. John Hitchman | 228 |
| The Human Mind, considered in some of its Medical Aspects | 246 |
| Supposed Demoniacal Possession | 262 |
| Insanity from Chloroform | 269 |
| Somerset County Lunatic Asylum | 271 |
| Miscellaneous Notices | 271 |
| The Editor's Portfolio | 274 |
| Books received for Review, &c. | 282 |

